# Utilisation, contents and costs of prenatal care under a rural health insurance (New Co-operative Medical System) in rural China: lessons from implementation

**DOI:** 10.1186/1472-6963-10-301

**Published:** 2010-11-01

**Authors:** Qian Long, Tuohong Zhang, Elina Hemminki, Xiaojun Tang, Kun Huang, Shengbin Xiao, Rachel Tolhurst

**Affiliations:** 1 Department of Public Health, University of Helsinki, Mannerheimintie 172, Helsinki, Finland; 2 School of Public Health, Peking University, Beijing, P.R. China; 3 National Institute for Health and Welfare, Lintulahdenkuja 4 (P.O. Box 30), FI-00271 Helsinki, Finland; 4 School of Public Health, Chongqing Medical University, No.1 Yixueyuan Road, Chongqing, P.R. China; 5 School of Public Health, Anhui Medical University, No. 81 Meishan Road Hefei, P.R. China; 6 School of Medicine, Xi'an Jiaotong University, No. 76 Yantaxi Road Shaanxi, P.R. China; 7 Liverpool School of Tropical Medicine, Pembroke Place, Liverpool, L3 5QA, UK

## Abstract

**Background:**

In China, the New Co-operative Medical System (NCMS), a rural health insurance system, has expanded nationwide since 2003. This study aims to describe prenatal care use, content and costs of care in one county where prenatal care is included in the NCMS and two counties where it is not. It also explores the perceptions of stakeholders of the prenatal care benefit package in order to understand the strengths and weaknesses of the approach in the context of rural China and to draw lessons from early implementation.

**Methods:**

This study is based on the data from a cross-sectional survey and a qualitative investigation conducted in 2009. A survey recruited women giving birth in 2008, including 544 women in RC County (which covered prenatal care) and 619, and 1071 in other two counties (which did not). The qualitative investigation in RC included focus group discussions with women giving birth before or after 2007, individual interviews with local policy makers and health managers, NCMS managers and obstetric doctors in township hospitals.

**Results:**

There were no significant differences in prenatal care use between RC County (which covered prenatal care) and other two counties (which did not): over 70% of women started prenatal visits early and over 60% had five or more visits. In the three counties: a small proportion of women received the number of haemoglobin and urine tests recommended by the national guideline; 90% of women received more ultrasound tests than recommended; and the out-of-pocket expenditure for prenatal care consumed a high proportion of women's annual income in the low income group. In RC: only 20% of NCMS members claimed the reimbursement; the qualitative study found that the reimbursement for prenatal care was not well understood by women and had little influence on women's decisions to make prenatal visits; and several women indicated that doctors suggested them taking more expensive tests.

**Conclusions:**

Whether or not prenatal care was included in the NCMS, prenatal care use was high, but the contents of care were not provided following the national guideline and more expensive tests were recommended by doctors. Costs were substantial for the poor.

## Background

Prenatal care aims to identify and address potential risk factors for adverse pregnancy outcomes in order to reduce pregnancy-related mortality and morbidity [1]. Both the accessibility and quality of care are important to be able to make prenatal care effective. Evidence-based practise guidelines and affordable costs are essential for achieving good quality of prenatal care and avoiding dropout from the care [2,3].

The new World Health Organization (WHO) prenatal care model recommends a minimum of four visits and provides detailed instructions on the basic components of care for the four-visits for both industrialised and developing countries [4]. Costs of accessing prenatal care including travel costs, service fees and test costs have been identified as a barrier to use care [5]. Hence, including a maternity benefit package into health insurance schemes or providing free care at the point of use has been advocated [6]. In 2001, in developing countries an estimated 68% of women had at least one prenatal visit [7]. Often, the prenatal care fails to meet the standards recommended by WHO due to lack of human resources, equipment and supplies [8].

In China, systematic maternal health care was introduced in the 1980 s. The Chinese Ministry of Health issued a national guideline, the updated version of which recommends starting prenatal visits within the first trimester of pregnancy (< = 12 gestation weeks) and having at least five prenatal visits [9]. It also describes the contents of care for each visit. There is significant disparity in prenatal care use between rural and urban areas. However, the national health services survey data in rural areas from 1993 to 2008 show an increase in early utilisation of prenatal care from 24% to 63% and in the proportion of women having five or more prenatal visits from 11% to 44% [10]. We found only one published study on the content of prenatal care, which shows only 50% of rural women had received components recommended [11].

In rural China, financial constraints have been proposed as the main cause for the under-use of health care in general [12]. Our previous study in western rural areas found that low income women were less likely to have five or more visits than women in the medium and high income groups in 2007 [13]. There is also evidence that the means of payment has an association with prenatal care use. Women participating in a health insurance scheme covering the cost of obstetric care made more prenatal visits than non-insured women [14].

In the mid 1950 s, a collectivism-based rural health insurance system was established, called Co-operative Medical System (CMS) which covered the cost of medical and obstetric care. With the transition from a collective economy to a market system at the end of 1970 s, the CMS collapsed leaving around 90% of the rural population uninsured in the 1990 s [15]. Since 2002, the Chinese government has given a high priority to re-establishing a new rural health insurance system, the New Co-operative Medical System (NCMS), which focuses on reducing risks of catastrophic costs of healthcare. The NCMS is organized, guided and supported by central and local governments and involves voluntary participation with a flat rate premium [16]. It operates at the county level and schemes vary across the counties. In 2007, the overall participation rate of total rural residents was 86% [17]. To our knowledge, in most counties the NCMS includes a maternity benefit package providing either a proportional or fixed amount of reimbursement for facility-based delivery, both for vaginal and caesarean delivery. Only a few counties have included prenatal care into the NCMS maternity benefit package.

This study aims to describe prenatal care use, content and costs of care in one county where prenatal care is included in the NCMS and two counties where it is not. It also explores the perceptions of stakeholders on the prenatal care benefit package in order to understand the strengths and weaknesses of the approach in the context of rural China and to draw lessons from early implementation.

### Study areas

This study is a part of the project 'Structural hinders to and promoters of good maternal care in rural China-CHIMACA (015396)' funded by the European Commission. One municipality and two provinces of western and central China were selected, representing relatively less developed areas of China. In each site, one or two counties were selected on the basis that there were no other maternal health care improvement programs in the county, they represented less developed or average socio-economic level areas of the study sites and local health facilities were willing to and capable of participating in the project. One county from each study site which did not have a financial intervention in prenatal care designed and implemented as part of the CHIMACA project - in total three counties - were included in this paper.

RC County is located in Chongqing Municipality in south-western China. In 2005, GDP per capita was RMB 8,999. Since 2007, part of prenatal care including some laboratory tests listed in the national guideline has been included in the NCMS. If their family is enrolled in the NCMS, women can claim reimbursement for specific tests, up to a maximum of RMB 100. A fixed amount of reimbursement for facility-based vaginal delivery and proportional reimbursement for caesarean delivery were provided from 2005 to 2007. From 2007 onwards RMB 400 reimbursements have been given for both vaginal and caesarean delivery.

XC County in Anhui province in central China had GDP per capita of RMB 9361 in 2005 and LT County in Shaanxi province in north-western China had RMB 5356. In both counties, the NCMS provided a fixed amount of reimbursement to facility-based delivery from 2007, but prenatal care is not included in the NCMS.

Rural areas can be categorised into three levels in terms of how restrictive the implementation of family planning policy is: the most restricted areas, secondary restricted areas and flexible areas. RC County is in the most restricted area where births to women having more than one child without permission from local family planning authority or having a child without legal marital status are considered as unauthorised births. XC and LT County are in the secondary restricted area where women are allowed to have a second child if the first one is a daughter. In the three counties, women having an unauthorised pregnancy cannot benefit from the maternity benefit package in the NCMS. Care for any complications during delivery is covered by another section of the NCMS, either for authorised or unauthorised pregnancy. Neither preventive nor curative healthcare for the newborn is included in the benefit package.

## Methods

Both quantitative and qualitative methods were used in this study.

### Quantitative study

A cross-sectional survey was carried out in all townships of each county at the end of 2008 and early 2009. One third of the villages in each township were randomly selected from a list stratified by the population size and distance to the township hospital. The target study populations were women having given birth from March to December in 2008 in XC County and from April to December in 2008 in RC and LT County. Women in XC County were identified from the family planning register and women in RC and LT County were identified from the health system register. The samples were 1023 in XC, 1029 in RC, and 1428 women in LT County. A structured questionnaire was used to collect information on the general demographic and socioeconomic background, knowledge of the maternity benefit package in the NCMS, utilisation of maternal health care and expenditures. Interviewers including researchers and Master's students from local medical universities were trained in data collection. The response rates were 61% in XC, 54% in RC and 75% in LT County. The primary reasons for non-response were similar in the three counties, including wrong address, no-one at home on the day of the survey, or the women being a rural-to-urban migrant.

For this paper, two indicators of prenatal care use were chosen: starting prenatal care within 12 gestation weeks and making five or more prenatal visits. The content of care provided was compared to the national guideline with regard to: advice on nutrition, avoiding alcohol, smoking and hazardous substances during pregnancy; routine blood pressure measurement and fetal heart monitoring; haemoglobin tests (three tests recommended); urine tests (two tests recommended); and ultrasound test (one test recommended). In addition, the out-of-pocket expenditure for prenatal care (reported medical expenditure during the prenatal visits) as a percentage of women's annual income was used as an indicator to evaluate affordability of care to rural women [18,19].

### Qualitative study

Qualitative investigation in RC County (which included prenatal care in the NCMS) was used to gain an understanding of stakeholders' perceptions of the inclusion of prenatal care and their views of the impact of this on prenatal care use and services provision. Qualitative data were drawn from a wider study of maternal health services and collected in 2009. The interviewers were trained in data collection skills and ethical approaches, including confidentiality. Focus Group Discussions (FGDs) were conducted with two groups of five women who had an authorised birth: one group gave birth before 2007 and one group gave birth after 2007. Semi-structured individual interviews were conducted with: 1) three key informants who are responsible for maternal health and the NCMS at the county level; 2) twelve health providers (health managers, NCMS managers and obstetric doctors from township hospitals). The trustworthiness of data was assured by triangulating findings from different respondents and methods and discussion of preliminary findings with local policy makers and maternal health care managers.

### Data analysis

Cross tabulation was used to show timing, frequency and the content of prenatal care in the three counties. Statistical differences in prenatal care use by the number of live births and contents of care by county were tested by the Chi-square test. Women's annual income was calculated as self-reported gross household income divided by the number of family members. Women were then grouped into three income categories (low, medium and high), each containing a third of the respondents. The out-of-pocket expenditure for prenatal care as a percentage of women's annual income was calculated in the different income groups in each county.

The qualitative data was analysed using the 'frame-work approach' [20]. An analytical framework was developed, based on the topic guide and categories emerging from the transcripts, and applied to the data to identify themes. All qualitative data was coded, sorted and classified using Maxqda2 software based on the framework. Charting was used to identify common or divergent perceptions and explanations were developed.

### Ethical considerations

The survey obtained the approval of the International Centre for Reproductive Health (ICRH), Ghent University. Ethical approval for the qualitative study was obtained from the Research Ethics Committee at the Liverpool School of Tropical Medicine. Local approval was obtained from the ethical committee of Anhui Medical University, Chongqing Medical University and Xi'an Jiaotong University. All of the data were collected with the informed consent of participants prior to their participation in the study. Views from these respondents have been anonymised in order to protect confidentiality.

## Results

### Description of prenatal care benefit package in the study counties

In RC, prenatal visits were free for all pregnant women living in this county (including authorised and unauthorised pregnancies). Free prenatal care included a visit to a health professional to have a physical check-up (routine weight and height measurement, fundal height, fetal abdominal circumference, blood pressure measurement, fetal heart monitoring) and health consultation and education related to pregnancy. This care has been paid for as a package totalling RMB 183 per pregnancy by the county government, since 2007. In 2007, the NCMS provided a fixed amount of reimbursement for a number of tests recommended by the national guideline, up to a maximum of RMB 100. Table 1 shows the charges for tests that were included in the NCMS at county and township level hospitals. The amount of reimbursement as a percentage of the hospital charges varied from 44% to 100% at township level; and from 33% to 100% at county level. In addition, women had to pay fully for pregnancy-related treatment and drugs. One health manager interviewed estimated that the expenditures for treatment and drugs per pregnancy varied from less than RMB 100 to over RMB 1000.

**Table 1:** Description of hospital charges (RMB) for prenatal care, by hospital level and the NCMS coverage in RC County in 2008

	Prenatal care included in RC			Prenatal care not included in XC		Prenatal care not included in LT	
	
	County	Township	NCMS coverage	County	Township	County	Township
Visit to doctor	Free	Free	No	Charged	Charged	Charged	Charged

Health consultation	Free	Free	No	Free	Free	Free	Free

Laboratory tests ^(a)^							

*Routine blood (×3)	11	9	6 (55~67%)	15	15	8	6

*Routine urine (×2)	9	9	4 (44~44%)	10	10	6	4

Blood type	8	5	5 (63~100%)	15	15	5	4

*Routine vaginal secretion	3	3	3 (100~100%)	5	2	5	/

*Liver function	40	34	18 (45~53%)	20	20	35	15

*Kidney function	23	12	8 (35~67%)	17	17	45	35

Hepatitis B	4	4	4 (100~100%)	5	5	35	25

Ultrasound	41/88**	41	25 (61/0~61%)	35	40	25	20

Coagulating time	33	13	11 (33~85%)	2	2	30	/

Referral	Charged	Charged	No	Charged	Charged	Charged	Charged

Other treatment	Charged	Charged	No	Charged	Charged	Charged	Charged

Source: Institutes data and policy documents issued by Chongqing Municipality Health Bureau.

County: county level hospital; Township: township level hospital.

NCMS coverage: the NCMS reimburses three routine blood tests, two routine urine tests and one of any other tests. The percentage in parentheses is the reimbursement as a percentage of county and township hospitals charges.

(a) Explanation of laboratory tests:

Routine blood test is to examine red and white blood cells and haemoglobin level.

Routine urine test is to check the levels of sugar and protein.

Routine vaginal secretion test is to check the colour and HP of vaginal secretion and test for bacteria.

Liver function test is to test GPT and GOT.

Kidney function test is to test BUN and Cr.

** The cost of ultrasound test for white-black image (RMB 41) and colour image (RMB 88). The latter is not covered by the NCMS.

/Routine vaginal secretion test and coagulating time test are not available at township hospital in LT County.

In XC and LT County, visits to a doctor to get information were free of charge, but otherwise women had to pay fully for prenatal care. In XC County, to have a physical check-up by a doctor cost about RMB 10 for the first visit, then RMB 3 for each of the following visits in both county and township level hospitals. In LT County, physical check-up cost RMB 8 at county level hospital and RMB 5 at township level hospital for each visit. XC County had the highest charge for routine blood and urine tests compared to RC and LT County; the charge for an ultrasound test was the highest in RC, followed by XC and lowest in LT County (Table 1).

### Women's socio-economic characteristics and knowledge of prenatal care benefit package

The number of respondents was 544 in RC, 619 in XC and 1071 in LT County. On average, women's annual income was the lowest in LT, followed by RC and the highest in XC County (Table 2). Women were well educated in LT: 91% of women had secondary education or higher, and this was 77% in RC and 65% in XC County. In the three counties, around 40% of women had two or more children. Most of the families participated in the NCMS. When we asked 'do you (NCMS member) know whether you can claim reimbursement for prenatal care? ', less than half of women answered 'yes' in RC County. A small proportion of women in XC and LT County thought that prenatal care was covered by the NCMS, although it was not. 40% and 31% of women in RC and LT, but only 3% in XC County had unauthorised births based on women's response. The low rate in XC County was probably due to women being identified for interview from the family planning register, which did not include many unauthorised births.

**Table 2:** Women's socio-economic characteristics and knowledge of prenatal care benefit package by county, women giving birth in 2008

	RC N = 544	XC N = 619	LT N = 1071
Women's annual income (Mean ± SD RMB)*	4397 ± 4212	6884 ± 5818	3152 ± 2469
Secondary education or higher % (N)†	77.1 (405)	65.1 (403)	91.2 (934)
Having two or more children % (N)	39.2 (213)	36.0 (223)	41.2 (441)
NCMS membership % (N)‡	71.2 (384)	82.3 (507)	83.8 (895)
Know of prenatal care benefit package % (N)§	48.5 (184)	1.2 (6)	5.0 (45)
Having unauthorised births % (N)	39.7 (216)	2.9 (18)	30.7 (329)

* Three women in RC with missing values;

† Nineteen women in RC and forty-seven women in LT with missing values;

‡ Five women in RC, three women in XC and three women in LT with missing values;

§ Only women who were NCMS members were asked; five women in RC with missing values.

The qualitative study in RC County investigated women's and obstetric doctors' knowledge about the prenatal care included in the NCMS. The FGDs with women having authorised births after 2007 showed that most of them knew that they could claim up to RMB 100 in reimbursements for prenatal care expenditure. However, almost all of them did not know which services were covered by the NCMS. Furthermore, some showed a distrust of doctors' decisions in implementing the benefit package.

' *We do not know (what could be reimbursed) at all. If doctors said you could get only RMB 50, then you just get RMB 50 of reimbursement. It depends on the decision of doctors, not ours*.' (Woman giving birth after 2007, FGD)

Almost all township obstetric doctors interviewed knew which tests were covered by the NCMS. Most of them could not remember how many of each eligible test could be reimbursed. A minority did identify a specific number of tests that could be reimbursed - e.g. one blood or urine test and two or three ultrasound tests.

### Timing and frequency of prenatal visits and perception of the impact of the NCMS on utilisation

Only 1% or less than 1% of women in the three counties had no prenatal visit. A large proportion of women started prenatal visits early and made five or more visits; over half of the women used prenatal care at a county level hospital. Women having two or more children were significantly less likely to make early and adequate prenatal visits than women having only one child (Table 3).

**Table 3:** Use of prenatal care by the number of live births and county, women giving birth in 2008, % (N)

	Start of prenatal visit ≤ 12 weeks †			5+ prenatal visits ‡		
	
	RC	XC	LT	RC	XC	LT
1^st ^child	78.1 (253)	78.5 (307)	81.2 (509)	75.3 (244)	73.7 (289)	67.8 (421)
2+ children	69.0 (138)*	70.0 (150)*	67.4 (289)**	64.0 (130)**	57.1(124)**	53.4 (225)**
Total	74.6 (391)	75.4 (457)	75.6 (798)	70.4 (375)	67.5 (413)	61.2 (646)

† Twenty women in RC, thirteen women in XC and fifteen women in LT with missing values;

‡ Eleven women in RC, seven women in XC and fifteen women in LT with missing values;

P-value refers to the difference in prenatal care use between the number of live births;

* P < 0.05; ** P < 0.01

The qualitative study in RC County explored the perception of stakeholders about the impact of the prenatal care coverage in the NCMS on utilisation. One leader at the county level said that prenatal care was included in the NCMS with the aim of improving prenatal care use through a financial incentive. He believed that this would be effective. In addition, he explained that the operation of the NCMS had to follow the national policy; hence, it was reasonable that the NCMS did not provide a subsidy to women having unauthorised pregnancies. On the other hand, other leaders at the county level perceived a problem with excluding women having unauthorised pregnancies who were less likely to use prenatal care. This divergence in opinion was also found among the township health managers, NCMS managers and obstetric doctors.

*'RMB 100 reimbursement for prenatal care can be an incentive to rural women and encourage them to visit doctor regularly. It must have this impact.... Our policy cannot be against national family planning policy. If the NCMS also provides reimbursement to women having unauthorised birth, I think that it is not reasonable. After all, those women are a minority. Even if the policy has its limitation, this is small.' *(Leader of the county level)

*'To achieve further reduction of maternal and infant mortality, we have to give more concern to those having unauthorised births. They are a high risk group and are not a small proportion in rural China. Usually, they are not supported by maternal health policy. It is very difficult to trade off between national family planning policy and other maternal health policy. But if we continue to ignore this group, it is hard to achieve our goal (reduction of maternal and infant mortality).' *(Leader of the county level)

Most of the women in FGDs who had authorised births made five or seven visits according to the doctors' suggestion. All of them said that the reimbursement for prenatal care was better than nothing, but it did not influence their decision to make prenatal visits. They stressed that their reason for making prenatal visits was to ensure own and their baby's safety.

### Content of care reported by women and providers' perceptions of the impact of the NCMS on services provision

In the survey, women reported the content of prenatal care they received. The highest proportion of women who received advice for safe pregnancy was found in RC, followed by XC and the lowest in LT County (Table 4). The national guideline recommends blood pressure measurement and fetal heart monitoring for each visit. In this study, the appropriate levels of care were measured by having three or more examinations, according to the guideline. The percentage was high in the three counties, at over 80%. In RC, the proportion of women having no haemoglobin and urine test was higher; and having the number of tests recommended (three haemoglobin tests and two urine tests) was lower than other two counties. The differences are statistically significant. Only a small proportion of women received a haemoglobin test three times, even in XC and LT County. In the three counties, a very high proportion of women had two or more ultrasound tests. Over half of women in XC had four or more tests, much higher than that in RC and LT County.

**Table 4:** The content of prenatal care by county, women giving birth in 2008, % (N)

	RC	XC	LT	P
Advice on nutrition during pregnancy given ^a^	91.2 (485)	85.1 (521)	77.4 (816)	<0.01
Advice on avoiding alcohol, smoking and hazardous substances given ^b^	90.1 (482)	82.4 (504)	69.0 (726)	<0.01
Blood pressure measurement ^c^				
0	3.2 (17)	1.6 (10)	1.8 (19)	0.13
*3+	81.1 (426)	84.5 (517)	82.5 (869)	
Fetal heart monitoring by stethoscope ^d^				
0	2.5 (13)	2.1 (13)	1.7 (18)	0.58
*3+	83.2 (435)	84.0 (514)	83.3 (874)	
Haemoglobin test ^e^				
0	32.2 (170)	10.0 (61)	21.4 (225)	<0.01
*3+	8.1 (43)	22.1 (135)	20.8 (219)	
Urine test ^f^				
0	19.9 (105)	6.2 (38)	6.9 (72)	<0.01
*2+	34.3 (181)	69.6 (426)	67.4 (707)	
Ultrasound screening ^g^				
0	0.9 (5)	0.3 (2)	0.5 (5)	<0.01
*1	11.5 (61)	2.8 (17)	6.6 (70)	
2	30.9 (164)	15.2 (93)	21.2 (224)	
3	29.2 (155)	28.8 (176)	37.0 (391)	
4+	27.5 (146)	52.9 (324)	34.7 (366)	

^a ^Twelve women in RC, seven women in XC and seventeen women in LT with missing values;

^b ^Nine women in RC, seven women in XC and nineteen women in LT with missing values;

^c ^Nineteen women in RC, seven women in XC and eighteen women in LT with missing values;

^d ^Twenty-one women in RC, seven women in XC and twenty-two women in LT with missing values;

^e ^Fifteen women in RC, seven women in XC and nineteen women in LT with missing values;

^f ^Sixteen women in RC, seven women in XC and twenty-two women in LT with missing values;

^g ^Thirteen women in RC, seven women in XC and fifteen women in LT with missing values;

* Recommended number of test in terms of the national guideline

P-value refers to the difference in the content of prenatal care between the counties

The qualitative study investigated women's experiences of making prenatal visits and explored the perceptions of health care providers on the impact of including prenatal care in the NCMS on the service provision. Most women in the FGDs reported having one haemoglobin test and urine test and two or three ultrasound tests. Almost all the women said that they followed doctors' suggestions to take tests. Several women indicated that doctors suggested them taking more expensive tests, which are not included in the benefit package.

' *When I had prenatal visit at county hospital, the doctor said that ultrasound screening with colour image would show clearly. It was expensive. But I did it.' *(Women giving birth before 2007 in FGD)

Most of the township health managers and obstetric doctors thought that the inclusion of prenatal care in the NCMS would be helpful to convince women to take necessary tests, but they did not perceive any impact on their provision of the services:

' *There is no change in service provision before and after the implementation of prenatal care benefit package...We followed the rule to provide services... Generally speaking, the tests included in the benefit package are essential. Now, some of them could be reimbursed. That benefits women. There was no influence to our hospital*.' (Township health manager)

### Cost of prenatal care

The survey in RC found that only 20% of the NCMS members claimed any reimbursement for prenatal care and the amount varied from RMB 16 to 100. The major reason of non-reimbursement was women having unauthorised pregnancies (70%). In addition, it was partly due to women not knowing the procedure for reimbursement. For example, they did not know they needed to keep receipts of expenditures. In RC, the mean out-of-pocket expenditure for prenatal care in each income group was lower than the other two counties. The out-of-pocket expenditure for prenatal care was 8% of women's annual income in RC, which was the same in XC, and was the highest in LT County (17%). In the low income group, this proportion was much higher than that in the medium and high income groups (Table 5).

**Table 5:** The out-of-pocket expenditure for prenatal care by income group and county in 2008

	Out-of-pocket expenditure (1)			Women's annual income (2)			Out-of-pocket expenditure, % of income (3) = (1)/(2)		
	
	RC	XC	LT	RC	XC	LT	RC	XC	LT
Low	320	483	528	1241	2287	1311	25.8	21.1	40.3
Medium	313	557	522	3896	6011	2806	8.0	9.3	18.6
High	399	601	608	9332	13586	5898	4.3	4.4	10.3
Total	340	544	550	4397	6884	3152	7.7	7.9	17.4

Women in FGDs reported expenditure for prenatal care ranging from RMB 200 to 500. Most of the women thought that it was worth spending money on prenatal care in order to ensure their baby's safety, although some of them felt it was a financial burden.

'*My family's condition (economic situation) is not good. The transportation alone cost around RMB 100. But I have no choice. I have to visit hospital in order to ensure the baby's safety. That is the most important thing*.' (Women giving birth after 2007 in FGD)

Township NCMS managers and obstetric doctors indicated they perceived that the amount of reimbursement for prenatal care was small. They added that one reason for this was that the criteria for the reimbursement were set up based on low technology approaches (e.g. taking blood samples and analysing samples by hand). However, health facilities used advanced equipment to test and consequently charged higher prices even for essential tests. In addition, many women did not attain the whole RMB 100 reimbursements available, due to either losing part of the receipts or not receiving all the tests recommended by the package. One township NCMS manager estimated that each pregnant woman only gained about RMB 40-50 reimbursement for prenatal care.

## Discussion

Whether in the county that included prenatal care in the NCMS, or counties that did not, a substantial percentage of rural women made early and five or more prenatal visits. However, in the study counties, some basic components of care were not provided according to the national guideline; instead, ultrasound test was performed the most frequently. A low proportion of the NCMS members claimed reimbursement: the main reason for not claiming was due to the care being sought for unauthorised pregnancies. In the low income group, the out-of-pocket expenditure for prenatal care accounted for a very high proportion of women's annual income, especially in the relatively poor county.

In this study, there were several limitations. Firstly, data on the content of prenatal care received by women and costs of care are subject to recall bias, though a one-year recall is unlikely to cause serious bias. Secondly, the non-response rates are around 40% in study counties. One of the major missing reasons was women being absent due to rural-to-urban migration. Those migrant women might make some or all of their prenatal visits at rural facilities, but they are likely to differ from rural resident women. We also did not have information about other non-respondents to assess if they differed from respondents on any important variables. In addition, the participants in focus group discussions were recruited by township doctors. Those women might have more contacts with doctors and thus had better awareness of prenatal care use. Generally, the study design and descriptive statistical analyses in this paper cannot be used to draw conclusions on the causality between availability of the prenatal care package in the NCMS and the utilisation of care. The results should therefore be viewed as preliminary and generalisations should be made with caution.

In the three counties, the proportions of women seeking early and adequate prenatal care were higher than national average level in rural China in 2008 [10] and reported levels in other developing countries [7,21], although the definition of early and adequate prenatal care use varied from those studies. This suggests that in general women's awareness and acceptance of the need for prenatal care use was relatively high. This may be associated with the high level of education in study counties [13]. Compared to women having only one child, women having two or more children were less likely to use prenatal care, which is consistent with other studies in China and worldwide [5,22].

Prenatal care as part of an insurance benefit package has been successful in increasing the number of visits in the Philippines and some African countries [23,24], although in the latter study its effectiveness depended on the context in terms of general prenatal care costs and utilisation rates. In our study, part of prenatal care included in the NCMS also aimed to improve prenatal care use, but its implementation was affected by several factors. The first is a factor specific to China: women having unauthorised pregnancies could not benefit from this package because of the national family planning policy (known as the 'one-child policy'). The qualitative study found that the prenatal care included in the NCMS did not influence the decision to make prenatal visits among women having only one child since the demand was high in this group. By contrast, women having unauthorised pregnancies used less prenatal care. Previous studies in rural China have reported a family financial strain created by the substantial fine for unauthorised births that discouraged couples from purchasing both prenatal and obstetric care [25,26]. A financial benefit may encourage this marginal group to use prenatal care at the recommended time and tests. But there was divergence with the NCMS policy, creating a dilemma for maternal healthcare policy makers.

A second possible reason for the failure of the prenatal care benefit package to improve prenatal care use was that it was not well understood by the women. In our study, less than half of the NCMS members knew that prenatal care was included in the NCMS. Those women who knew that the reimbursement was available for prenatal care did not know the details of the policy and they showed distrust of doctors with regard to the amount of reimbursement that would be provided. A review on general health services delivery in China reported that many patients complained about unclear information about services they received and a few of them or their relatives had open conflict with health providers [27]. Studies in both industrialised and developing countries have found that good patient-provider interaction was associated with increased women's satisfaction [28,29], which was considered as one of the major factors influencing the use of prenatal care and the effect of care [30]. The lack of clear and effective communication suggested in this study may not only undermine the provider-user relationship, but also potentially affect health seeking behaviour if patients are unwilling to seek care due to lack of trust. Lack of knowledge about benefits available and claim processes have also been identified as barriers both to uptake of general health services covered by health insurance and to successfully claiming against insurance by women in India [31] as well as other studies in China [32-34].

Insurance-related increase of unnecessary service provision has drawn attention from policy makers internationally. In Taiwan, the use of more expensive prenatal care services notably increased after the implementation of national health insurance [35]. Our study found that the NCMS provided reimbursement for the specific tests and number of tests recommended by the national guideline. This is a cost-containing measure as well as a quality control measure. However, the survey in the three counties found that the basic components of care, such as haemoglobin tests and urine tests, were not carried out following the guideline, and this was particularly so in the county having the prenatal care coverage in the NCMS; while 90% of women received ultrasound tests two or more times. We did not investigate the detailed knowledge of the guideline among health care providers. It may be that ultrasound test is seen as the most important channel to identify risk during pregnancy. However, our qualitative study indicated that some doctors recommended more expensive tests to women, which suggests that considerations other than clinical needs are influencing their provision.

Since health sector reforms were launched in mid-1980, the government allocation to health care has shrunk and the health care system has become heavily dependent on fee-for-services financing. A fee-for-services payment method acts as an incentive to health care providers to over-provide services [36]. Public health care has suffered from a lack of funding and effective guidance from the central and local government in China. Healthcare providers have little incentive to provide non-profit services. Instead, they recommend more or more expensive services than clinically necessary. A study in other areas of China reported that the frequent use of ultrasound tests was to generate more revenue for salaries and pensions of hospital staff [37]. The failure to follow the guidelines for providing prenatal care raises questions about the quality of care. Meanwhile, providing expensive and possibly unnecessary services also increases women's financial burden due to prenatal visits.

According to our results, in RC County, the out-of-pocket expenditure for prenatal care was lower than other two counties. However, given that only a small proportion of women claimed the reimbursement and the amount was small, the NCMS may make little contribution to the lower expenditure for prenatal care. The lower expenditure in this county may be attributed partly to the fact that free prenatal visits are offered for all pregnancies funded by the county government and partly to the fact that fewer tests including some basic tests and ultrasound test were performed than other two counties. In the three counties, the out-of-pocket expenditures as a proportion of women's annual income in the low income group were high. When indirect costs are added including transportation for prenatal visits, the expenditures are substantial for the rural poor. Including prenatal care in the NCMS offers an opportunity to reduce the financial burden caused by prenatal visits, especially for the poor, but our study suggests that greater attention to the relationship between financial protection, quality of provision and provider payment mechanisms is necessary to make this opportunity a reality.

The lessons from our study have important policy implications. Since 1994, the orientation of the Chinese family planning has shifted from a focus on birth control to an integration of birth planning with quality and safety of reproductive health care, poverty alleviation and economic development [38]. The NCMS, a rural health insurance scheme, is intended to contribute towards addressing specific health needs of the target population. Hence, it has to consider how to address current conflicts between national family planning policy and NCMS policy in order to protect health of both authorised and unauthorised pregnancies. Moreover, where inclusion of pre-natal care in the NCMS is adopted, dissemination of the benefit package of the NCMS scheme and reimbursement procedure should be strengthened. In addition, either lack of related knowledge or revenue driven behaviour contribute to questionable quality of prenatal care in rural areas. Further research on factors influencing provider behaviour is needed in order to generate evidence for intervention development. Although one factor affecting the implementation of rural health insurance as a mechanism for improving utilisation and quality of provision was unique to China (the one-child policy), the study also offers lessons for international policy and research. In particular the study illustrates the importance of taking a health systems approach to evaluation that links utilisation and provision of services with the wider context of policy, patient information and demand, and provider motivations.

## Conclusion

In rural China, prenatal care use was high, but the contents of care provided did not follow the national guideline and more expensive tests were recommended by doctors. A low proportion of NCMS members claimed the reimbursement available, with the most frequent reason for not claiming being the unauthorised status of the pregnancy for which care was sought. The cost of pre-natal care was substantial for rural poor whether or not it was included in the NCMS benefit package. Further efforts to increase access to affordable, quality prenatal healthcare are therefore needed.

## Conflict of interest statement

The authors declare that they have no competing interests.

## Authors' contributions

QL was involved in data collection and management, and conducted the analysis, interpreted the findings and drafted the manuscript. TZ participated in qualitative study design, contributed to the interpretation of the findings and commented on the article. EH initiated the study concept and contributed to interpret findings and comment on the article. XT, KH and BX participated in the data collection and management. RT participated in qualitative study design, interpreting the findings and writing the article. All authors read and approved the final manuscript.

## Pre-publication history

The pre-publication history for this paper can be accessed here:

http://www.biomedcentral.com/1472-6963/10/301/prepub
